# Nicotine dependence based on the tobacco dependence screener among heated tobacco products users in Japan, 2022–2023: The JASTIS study

**DOI:** 10.1002/npr2.12512

**Published:** 2025-01-09

**Authors:** Takuma Kitajima, Takashi Hisamatsu, Hideyuki Kanda, Takahiro Tabuchi

**Affiliations:** ^1^ Department of Public Health Okayama University Graduate School of Medicine, Dentistry and Pharmaceutical Sciences Okayama Japan; ^2^ Division of Epidemiology, Department of Health Informatics and Public Health, School of Public Health Tohoku University Graduate School of Medicine Sendai Japan

**Keywords:** cross‐sectional survey, heated tobacco products, logistic regression, nicotine dependence, tobacco dependence screener

## Abstract

Heated tobacco products (HTPs) are nicotine‐containing products similar to cigarettes and are widely used in Japan. However, there has been insufficient research on nicotine dependence associated with HTP use. This study investigated the association of the types of individuals who smoked with the prevalence of nicotine dependence. We utilized data from the Japan Survey on Tobacco and Health (JASTIS). A total of 7969 participants who currently smokes was selected from the 2022 and 2023 survey respondents for the analysis. Nicotine dependence was defined as a score of 5 or higher on the Tobacco Dependence Screener (TDS). The prevalence of nicotine dependence was 43.0% (3473/8077) among all participants who smoked, 42.9% (1479/3447) among those who used cigarettes, 44.2% (760/1720) among those who used two products, and 43.0% (1206/2802) among those who used HTPs. The prevalence of nicotine dependence was statistically higher in the participants who used two products than in cigarettes (odds ratio [OR], 1.17; 95% confidence interval [CI], 1.04–1.33). When classified by temperature, participants who used of two products (high‐temp and low‐temp) and those using participants who used HTPs (high‐temp) had higher ORs for prevalent nicotine dependence (OR, 1.31 [95% CI, 1.14–1.51]) and (OR, 1.12 [95% CI, 1.00–1.25], respectively) compared to participants who used cigarettes. Additionally, the ORs for prevalent nicotine dependence increased with the number of tobacco sticks smoked per day. These results suggest that HTP use, particularly high‐temperature HTPs use, and a higher number of tobacco sticks smoked is associated with nicotine dependence.

## Graphical abstract

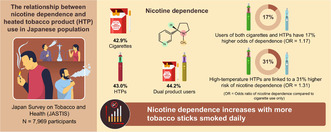

## 1 INTRODUCTION

Heated tobacco products (HTPs) are nicotine‐containing products, like cigarettes. They were launched in Japan in 2014 and spread rapidly.^1^, ^2^, ^3^ E‐cigarettes, which are common worldwide, are not popular in Japan because their sale is prohibited. This may be the reason that HTPs have spread widely in Japan. HTPs function by electrically heating tobacco leaves or their processed products using a special tool that generates smoke. In HTPs, the temperature at which tobacco is heated varies depending on the product.^4^ When smoking the low‐temperature heating type HTPs (low‐temp), do not heat tobacco directly, but rather heat the liquid (the main liquid ingredients are propylene glycol, vegetable glycerin, and fragrance) at 30–40°C to generate an aerosol that passes through a tobacco leaf or flavored tobacco capsule attached to the tip.^5^ The high‐temperature heating type HTPs (high‐temp), however, are products in which a special stick filled with tobacco leaves is inserted into a rechargeable device and heated at 200–350°C to produce an aerosol that is inhaled.^6^ The combustion temperature of cigarettes is 700–900°C. Both types require batteries and special cigarettes for heating, whereas those that do not directly heat the tobacco require a liquid that produces vapor.^7^ Studies have shown that 22.1% of male who smoke and 14.8% of female who smoke in Japan currently use HTPs.^8^

Despite these data, studies on the health hazards of HTPs, which have only recently gained popularity, are limited. Cigarette smoke contains over 5300 chemicals, including over 200 toxic substances and 50 carcinogens.^9^, ^10^ HTPs' emissions also contain harmful substances (aerosol generated by HTPs contains toxic substances such as nicotine, menthol, glycerin, diacetyl, acrolein, acetaldehyde, and glycidol),^11^, ^12^ although there has been no nationwide dose–response studies on nicotine dependence in HTPs in Japan.

In this study, we examined nicotine dependence among participants who used cigarettes, participants who used HTPs, and participants who used two products across Japan using nationwide data from the Japanese Internet Survey on Society and New Tobacco (JASTIS). Nicotine dependence is a crucial factor in estimating harmfulness because both HTPs and cigarettes contain nicotine.^13^ We also explored the differences in prevalence of nicotine dependence according to the heating type (low‐ or high‐temp) of HTPs and the number of tobacco sticks, that an individual smokes per day.

## 2 METHODS

### 2.1 Data source

**FIGURE 1:**
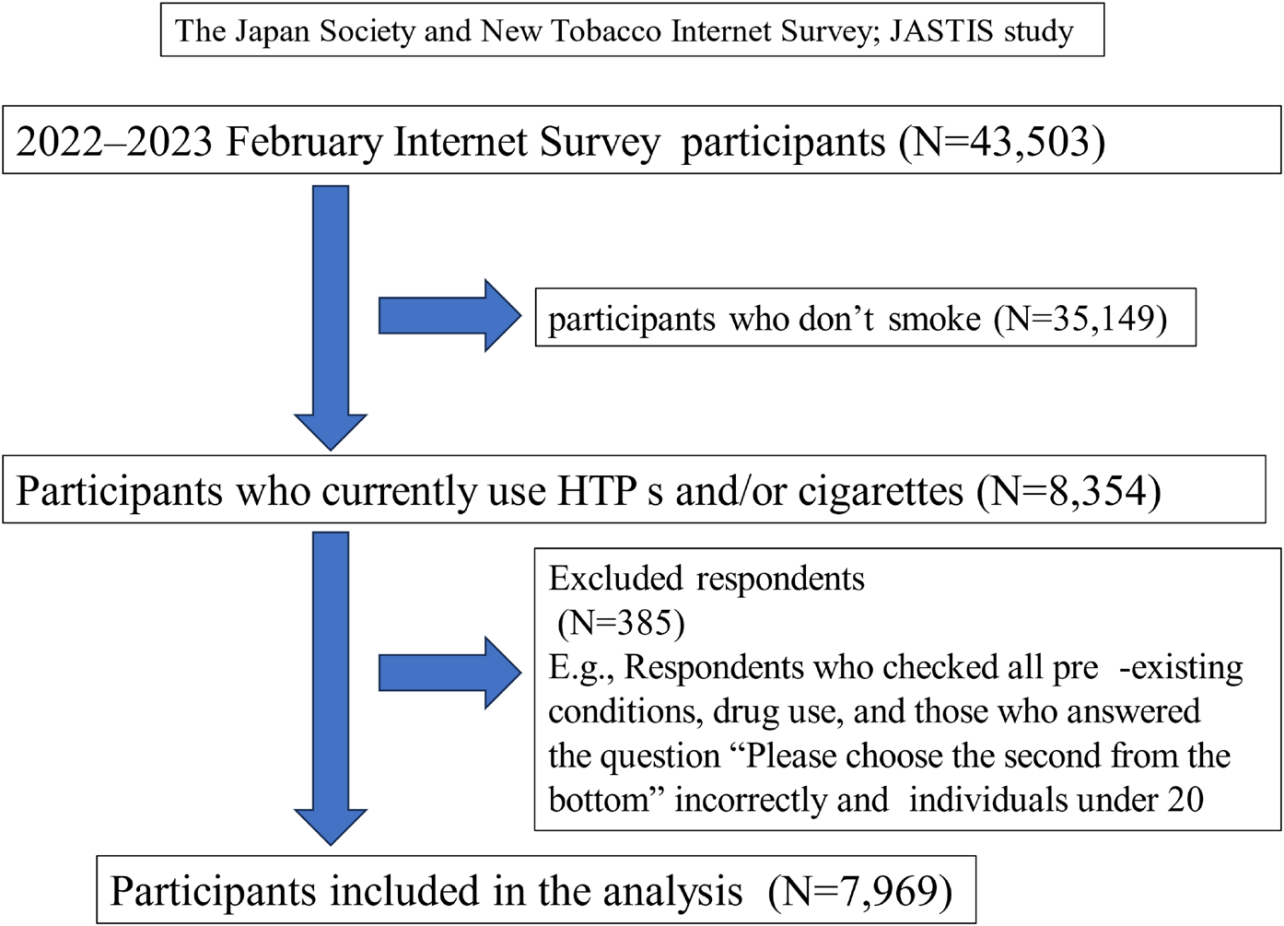
Flowchart of study participants.

The JASTIS was established in 2015 and conducts annual Internet‐based cross‐sectional surveys.^14^ This study employ a cross sectional longitudinal design, led by Dr. Tabuchi of the Department of Public Health at Tohoku University. Its data has been used by universities and research institutions nationwide. It aims to examine the use and perceptions of traditional tobacco products (i.e., cigarettes, HTPs, and e‐cigarettes) and the related socioeconomic indicators. The participants were recruited from a large survey panel operated by Rakuten Insight, a leading Japanese Internet research firm. The present study was based on the JASTIS data for 2022 and 2023 for individuals aged 15–82 years. A total of 8354 participants who currently smoked HTPs or cigarettes were selected from among the respondents (*n* = 43 503) in the 2022 (*n* = 33 000) and 2023 (*n* = 34 000) surveys. We excluded 385 respondents: those who checked all pre‐existing conditions, including drug use, and those who answered the question “Please choose the second from the bottom” incorrectly and individuals under 20. Figure 1 presents a flowchart of the derivation of the analysis. The final sample for analysis comprised 7969 respondents.

This study was conducted in accordance with the Ethical Guidelines for Life Science and Medical Research Involving Human Subjects.^15^ All study protocols were reviewed and approved by the Research Ethics Committee (Protocol Number 20084). Informed consent was obtained from all participants at the time of study entry by electronic means.

### 2.2 Measures

Nicotine dependence was defined as a score of 5 or higher on the Japanese version of Tobacco Dependence Screener (TDS) dependence test, which is a 10‐question test to determine the degree of dependence.^16^, ^17^, ^18^, ^19^ This screener was developed to diagnose nicotine dependence from a psychiatric perspective in accordance with the WHO's International Classification of Diseases, Tenth Edition (ICD‐10), and the revised third and fourth editions (DSM‐III‐R and DSM‐IV) of the American Psychiatric Association's “Guide to the Classification and Diagnosis of Mental Disorders.” Cronbach's alpha coefficients for the TDS ranged from 0.74 to 0.81 among male and female smokers, indicating a good internal consistency.^17^ This scale revealed acceptable construct validity, predictive validity, and screening performance based on psychiatric diagnosis criteria.^18^ Compared with the Fagerström test nicotine dependence, the TDS has a better screening performance for ICD‐10, DSM‐III‐R, and DSM‐IV diagnoses.^17^ These findings suggest that the TDS is a reliable and useful screening questionnaire for tobacco/nicotine dependence according to ICD‐10, DSM‐III‐R, and DSM‐IV. Participants who smoked were asked, “Do you currently smoke or use tobacco?” A participant who smokes was defined as a person who answered “sometimes” or “almost every day” to cigarettes, hand‐rolled cigarettes, Ploom Tech, Ploom S, Ploom X, IQOS, and Lil HYBRID. Cigarettes and hand‐rolled cigarette users were defined as the participants who used cigarettes. Ploom Tech, Ploom S, Ploom X, IQOS, and Lil HYBRID users were included in the participants who used HTPs, while those using both cigarettes and HTPs were included in the participants who used two products. We divided HTPs into two groups: the participants who used low‐temp HTPs for Ploom Tech and the participants who used high‐temp HTPs for Ploom S, Ploom X, IQOS, and Lil HYBRID. The number of tobacco sticks, smoked per day was calculated by asking, “How many days in the last 30 days did you smoke or use each of these cigarettes?” and “Approximately how many cigarettes do you use (smoke) in a day?” Information on participants' demographics (e.g., age, sex, household income, education, marital status, and alcohol consumption) was also collected using Internet‐based questionnaires.

### 2.3 Statistical analyses

Participants' characteristics were compared by type of smokers (participants who used cigarettes, participants who used two products, and participants who used HTPs) using the *χ* ^2^ test. We also examined the distribution of the number of tobacco sticks smoked per day. We estimated odds ratios (ORs) (95% confidence intervals [CIs]) for HTPs use (vs. cigarettes use) in relation to participants' characteristics. Furthermore, logistic regression analysis was used to examine the association between usage type (participants who used cigarettes as a reference) and nicotine dependence. We then used logistic regression analysis for nicotine dependence in relation to usage type, considering the differences in the HTPs' heating temperatures (i.e., participants who used cigarettes [as a reference]; participants who used two products [high‐temp, high‐temp and low‐temp, low‐temp]; and participants who used HTPs [high‐temp, high‐temp and low‐temp, low‐temp] and daily number of tobacco sticks [number smoked/day] [<10; 10–20; >20]). The adjusted model included sex (men/women), age categories (years: 20–29/30–39/40–49/50–59/60–69/70–82), annual household income (yen: less than 2 million/2–6 million/more than 6 million/don't know/don't want to say), daily alcohol consumption (2 drinks or less/3–6 drinks/7 drinks or more), educational attainment (high school graduate or less/vocational school graduate/college graduate or more), and marital status (married/never married/divorced or deceased). Two‐tailed tests were performed at the 5% significance level. All analyses were performed using the STATA statistical software (version 17.0; StataCorp LP, College Station, TX, USA).

## 3 RESULTS

Table 1 shows the characteristics of the participants who smoked according to usage type. Among the participants who smoked, 3447 (43.3%), 1720 (21.6%), and 2802 (35.2%) were participants who used cigarettes, participants who used two products, and participants who used HTPs, respectively. The majority of participants who smoked, 72.1%, were men. Compared with the participants who used cigarettes, the participants who used two products and participants who used HTPs had a higher prevalence of men and younger age groups, higher household income and alcohol consumption, and were more educated. The proportion of the participants who used two products was particularly high among never married groups.

**TABLE 1:** Participants' characteristics by usage types: The JASTIS study.

	Participants who used cigarettes	Participants who used two products	Participants who used HTPs	*p* value*
	*N* = 3447	*N* = 1720	*N* = 2802	
Gender				< 0.001
Men	2397 (69.5)	1332 (77.4)	2018 (72.0)	
Women	1050 (30.5)	388 (22.6)	784 (28.0)	
Age range (years)				< 0.001
20–29	335 (9.7)	502 (29.2)	695 (24.8)	
30–39	453 (13.1)	340 (19.8)	623 (22.2)	
40–49	732 (21.2)	351 (20.4)	663 (23.7)	
50–59	798 (23.2)	266 (15.5)	450 (16.1)	
60–69	665 (19.3)	211 (12.3)	285 (10.2)	
70–82	464 (13.5)	50 (2.9)	86 (3.1)	
Household income (yen)				< 0.001
Less than 2 million	332 (9.6)	163 (9.5)	249 (8.9)	
2–6 million	1407 (40.8)	629 (36.6)	1045 (37.3)	
More than 6 million	1036 (30.1)	680 (39.5)	1078 (38.5)	
Don't know	386 (11.2)	131 (7.6)	246 (8.8)	
Don't want to say	286 (8.3)	117 (6.8)	184 (6.6)	
Alcohol consumption				< 0.001
Fewer than 2 drinks	2450 (71.1)	1017 (59.1)	1826 (65.2)	
3–6 drinks	849 (24.6)	542 (31.5)	815 (29.1)	
More than 7 drinks	148 (4.3)	161 (9.4)	161 (5.7)	
Education attainment				< 0.001
Less than high school graduate	1232 (35.7)	485 (28.2)	843 (30.1)	
Vocational school graduate	699 (20.3)	294 (17.1)	545 (19.5)	
College graduate or higher	1516 (44.0)	941 (54.7)	1414 (50.5)	
Marital status				<0.001
Married	1965 (57.0)	923 (53.7)	1668 (59.5)	
Never	1017 (29.5)	632 (36.7)	833 (29.7)	
Divorced/dead	465 (13.5)	165 (9.6)	301 (10.7)	

*Note*: Values are expressed as the number of participants (%). The *χ* ^2^ test was used. *P value <0.05 for *χ* ^2^ test.

Table S1 shows the distribution of cigarettes and HTPs smoked per day. Table S2 shows the relevant characteristics for participants who used HTPs. [Table 1].

Table 2 shows the ORs for prevalence of nicotine dependence according to the usage type based on the logistic regression adjusted for sex, age, household income, alcohol consumption, educational level, and marital status. The prevalence of nicotine dependence was 43.0% (3473/8077) among all participants who smoked. The prevalence of nicotine dependence was 42.9% (1479/3447) among participants who used cigarettes, 44.2% (760/1720) among those who used two products, and 43.0% (1206/2802) among those who used HTPs. The prevalence of nicotine dependence was statistically higher in the participants who used two products than in the participants who used cigarettes (OR, 1.17; 95% confidence interval [CI], 1.04–1.33). There were no significant differences between the participants who used HTPs and participants who used cigarettes (OR, 1.08; 95% CI, 0.97–1.20).

**TABLE 2:** The odds ratios (ORs) for prevalent nicotine dependence by usage types.

	No. of nicotine dependence (%) / No. of participants	OR	95% CI	*p* value*
All participants who smoked	3445 (43.2) / 7969			
Participants who used cigarettes	1479 (42.9) / 3447	1.00	ref.	
Participants who used two products	760 (44.2) / 1720	1.17	1.04–1.33	0.010
Participants who used HTPs	1206 (43.0) / 2802	1.08	0.97–1.20	0.177

*Note*: Logistic regression analysis was adjusted for gender, age, household income, alcohol consumption, educational level, and marital status.

Abbreviations: CI, confidence interval; HTP, heated tobacco product.

* *P* value for logistic regression analysis.

Table 3 shows the ORs for prevalence of nicotine dependence by usage type based on the logistic regression adjusted for sex, age, household income, alcohol consumption, educational level, and marital status, considering the differences in the heating temperatures of HTPs. The ORs for the prevalence of nicotine dependence were higher in the high‐temp subgroups of both the participants who used two products and participants who used HTPs than in the participants who used cigarettes (OR, 1.31; 95% CI, 1.14–1.51 and OR, 1.12; 95% CI, 1.00–1.25, respectively). Compared with the participants who used cigarettes, there were no significant differences in the prevalence of nicotine dependence among the others such as those who used two products (high‐temp and low‐temp [OR, 0.99; 95% CI, 0.80–1.23] and low‐temp [OR, 0.90; 95% CI, 0.66–1.22]) and those who used HTPs (high‐temp and low‐temp [OR, 0.88; 95% CI, 0.65–1.19] and low‐temp [OR, 0.78; 95% CI, 0.56–1.09]).

**TABLE 3:** The odds ratios (ORs) for prevalent nicotine dependence by usage type considering differences in the heating temperature of HTPs.

	No. of nicotine dependence (%) / No. of participants	OR	95% CI	*P* value*
Participants who used cigarettes	1479 (42.9) / 3447	1.00	ref.	
Participants who used two products				
High‐temp	525 (47.0) / 1117	1.31	1.14–1.51	<0.001
High‐temp and low‐temp	163 (39.1) / 417	0.99	0.80–1.23	0.940
Low‐temp	72 (38.7) / 186	0.90	0.66–1.22	0.506
Participants who used HTPs				
High‐temp	1074 (44.1) / 2437	1.12	1.00–1.25	0.039
High‐temp and low‐temp	73 (36.3) / 201	0.88	0.65–1.19	0.404
Low‐temp	59 (36.0) / 164	0.78	0.56–1.09	0.142

*Note*: Logistic regression analysis was adjusted for sex, age, household income, alcohol consumption, educational level, and marital status.

Abbreviations: CI, confidence interval; HTP, heated tobacco product.

* *P* value for logistic regression analysis.

Table 4 shows the ORs for prevalence of nicotine dependence according to the number of tobacco sticks smoked per day based on the logistic regression adjusted for sex, age, household income, alcohol consumption, educational level, and marital status. The ORs for the prevalence of nicotine dependence were higher for a higher daily number of tobacco sticks smoked for each usage type. The ORs were higher for the group that smoked <10 products per day compared with those that smoked 10–20 and >20 products per day.

**TABLE 4:** The odd ratios (ORs) for prevalent nicotine dependence by number of tobacco sticks smoked per day.

	No. of nicotine dependence (%) /no. of participants	OR	95% CI	*p* values*
Participants who used cigarettes, number smoked/day				
<10	461 (31.1)/1480	1.00	ref.	
10–20	585 (48.9)/1196	2.11	1.80–2.47	<0.001
>20	433 (56.2)/771	2.87	2.39–3.45	<0.001
Participants who used two products, number smoked/day				
<10	258 (31.1)/830	1.04	0.86–1.26	0.693
10–20	248 (52.4)/473	2.56	2.07–3.17	<0.001
>20	254 (60.9)/417	3.67	2.92–4.61	<0.001
Participants who used HTPs, number smoked/day				
<10	435 (32.2)/1352	1.07	0.91–1.26	0.409
10–20	368 (45.9)/801	1.86	1.55–2.22	<0.001
>20	403 (62.1)/649	3.67	3.01–4.47	<0.001

*Note*: Logistic regression analysis was adjusted for sex, age, household income, alcohol consumption, educational level, and marital status.

Abbreviations: CI, confidence interval; HTP, heated tobacco product.

* *P* value for logistic regression analysis.

## 4 DISCUSSION

In this study, the prevalence of nicotine dependence was higher in the participants who used two products group than in the participants who used cigarettes group. Furthermore, the prevalence of nicotine dependence was also higher in the high‐temp participants who used two products and participants who used HTPs compared to the participants who used cigarettes, but this was not the case for the participants who used low‐temp. Furthermore, the prevalence of nicotine dependence increased with a higher daily number of tobacco sticks, smoked in each usage type. Participants who used HTPs were also found to be more likely to be male, younger, and heavier drinkers, similar to previous studies.^20^

The prevalence of nicotine dependence among participants who smoked in our study was 43%, which was not significantly different from those reported in previous studies. The prevalence of nicotine dependence has been previously estimated at 40%–70% among participants who smoked, although it varies by age, country, and measurement method.^21^, ^22^, ^23^, ^24^ For example, in 2019, American participants who used cigarettes (54.1% men; 40.2% aged 18–34 years; 29.0% aged 35–49 years; 69.8% non‐Hispanic White) reported a 56% prevalence of nicotine dependence.^24^ However, there have been few reports of nicotine dependence among participants who used HTPs. The present study is the first to clarify dependence on HTPs nationwide. Only one Japanese study on nicotine dependence among participants who used HTPs has found that participants who used HTPs, including participants who used two products and single users, were more dependent on nicotine (defined as the number of minutes after waking up before their first smoke) than those who only smoked cigarettes.^25^

The underlying mechanisms for the association with the prevalence of nicotine dependence in participants who used HTPs (i.e., participants who used two products or participants who used HTPs) remain unknown. Previous studies have shown that the number of tobacco sticks smoked increased after switching from cigarettes to HTPs, although they differ from the present study in that most participants who used HTPs are participants who used two products.^25^ In this study, the number of tobacco sticks smoked per day was higher in the participants who used HTPs than in the participants who used cigarettes (Table S1), and the number of tobacco sticks smoked was closely related to nicotine dependence. As shown in Table 4, for all smoking groups, the prevalence of nicotine dependence increased with the number of tobacco sticks smoked. Therefore, the prevalence of nicotine dependence in the participants who used HTPs was higher than that in the participants who used cigarettes because of the increase in the number of tobacco sticks smoked.

We also considered the following reasons for the increase in the number of tobacco sticks smoked in the participants who used HTPs compared with the participants who used cigarettes. First, participants who used HTPs may be less conscious of their surroundings when using HTPs than when using cigarettes. The revised Health Promotion Law in Japan regulates HTPs differently from cigarettes (e.g., public smoking is prohibited for cigarettes, but smoking is permitted in some areas for HTPs),^26^ and some participants who smoked may not think that HTPs cause harm to others through secondary smoke inhalation. However, some reports demonstrate that tobacco‐specific nitrosamine metabolites, which are biomarkers of health effects, have been detected in the urine of participants who never smoke exposed to HTP aerosols^27^ and symptoms such as sore throat, cough, chest pain, and mood disorders have been identified.^28^ Therefore, it might be a misconception to assume that HTP use does not necessitate consideration for the surrounding environment. Second, participants who smoked may think that HTPs are less harmful to their health than cigarettes. According to an Internet survey, many participants who used HTPs believe that using HTPs reduces their health risks more than when they use only cigarettes.^29^ Finally, participants who used HTPs may use more products than participants who used cigarettes because they seek nicotine more resolutely. In a study comparing the mainstream use of cigarettes and HTPs, the nicotine concentration in HTPs was lower than that in cigarettes, although there were differences between products.^30^ Additionally, people who become addicted to cigarettes begin using HTPs to reduce their frequency of use and smoking harm. Cigarette addicts may use HTPs, and therefore, a reversal of cause and effect may be present.

In this study, the participants who used high‐temp HTPs, but not the participants who used low‐temp HTPs, had a higher prevalence of nicotine dependence than the participants who used cigarettes. High‐temp HTPs tend to smoothly increase blood nicotine levels, whereas low‐temp HTPs cannot easily increase blood nicotine levels.^25^ Therefore, it is assumed that many of the users who use low‐temp HTPs may be originally less nicotine‐dependent or may have a shorter smoking history. This novel study clarifies nicotine dependence in HTPs using a nationwide survey. However, our study has some limitations. First, the number of tobacco sticks smoked was based on a self‐administered questionnaire, which may have led to underestimation. Second, the TDS is used to define nicotine dependence, but the questionnaire is designed to give higher scores to participants who smoke and who are conscious of quitting smoking, which may differ from actual nicotine dependence. For example, the TDS has questions: “Have you ever thought about quitting smoking cigarettes?” and “Have you ever smoked more cigarettes than you thought you would?” that are designed to help people quit smoking. For these questions, even those who smoke more cigarettes per day will not select “yes” if they are not conscious of quitting smoking, resulting in lower TDS scores.^19^ Third, Ploom Tech uses a capsule, which differs from high‐temperature HTP that uses a stick. Therefore, we are not sure if it is fair to count the number of tobacco sticks per day in the same way as other products, including cigarettes. Fourth, participants who used high‐temp HTP may have exhibited a high degree of nicotine dependence from the outset. Although an analysis of the ‘smoking history’ of the respondents (e.g., Brickman index) associated with each product would have been beneficial, this assessment was not feasible within the scope of this study. Finally, as this study involved an Internet survey conducted only in Japan. our results may not be generalizable to other ethnic populations. Therefore, larger‐scale prospective, multi‐country studies will be required to address the limitations of this study.

## 5 CONCLUSION

To the best of our knowledge, this is the first study to clarify the prevalence of nicotine dependence among participants who used HTPs based on a nationwide survey. High‐temp HTPs are associated with nicotine dependence and an increase in the number of tobacco sticks smoked. Thus, promoting smoking cessation initiatives among participants who used HTPs, and cigarettes is essential.

## AUTHOR CONTRIBUTIONS

Takuma Kitajima, and Takahiro Tabuchi designed the study. Hideyuki Kanda, and Takahiro Tabuchi supervised the study. Takuma Kitajima, and Takahiro Tabuchi developed the methodology. Takuma Kitajima created the dataset. Takuma Kitajima analyzed the data. Takuma Kitajima, and Takashi Hisamatsu wrote the first draft of the manuscript. Hideyuki Kanda, and Takahiro Tabuchi commented on the manuscript. All authors read and approved the final version. All authors confirm that they had full access to all the data in the study and accept responsibility to submit for publication.

## CONFLICT OF INTEREST STATEMENT

KK received a JMWH Bayer Grant (1 million Japanese yen) from September 1, 2017, to August 31, 2019.

## ETHICS STATEMENT

The protocol for this research project has been approved by a suitably constituted Ethics Committee of the institution and it conforms to the provisions of the Declaration of Helsinki. Institutional Review Board of the Osaka International Cancer Institute Approval No. 20084.

## CONSENT

Informed consent was obtained from all participants at the time of study entry by electronic means.

## APPROVAL OF THE RESEARCH PROTOCOL BY AN INSTITUTIONAL REVIEWER BOARD

The protocol for this research project has been approved by a suitably constituted Ethics Committee of the institution and it conforms to the provisions of the Declaration of Helsinki. Institutional Review Board of the Osaka International Cancer Institute Approval No. 20084.

## REGISTRY AND THE REGISTRATION NO. OF THE STUDY/TRIAL

No applicable.

## Supporting information


Table S1.
Table S2.

## Data Availability

The data for this study are available as Supporting Information.
