# Supplementary material for: Nicotine dependence based on the tobacco dependence screener among heated tobacco products users in Japan, 2022–2023: The JASTIS study
Source: Neuropsychopharmacol Rep. 2025 Jan 9;45(1):e12512. doi: 10.1002/npr2.12512 (PMC11711695; doi:10.1002/npr2.12512)
Supplement: Supplementary file 1 — Table S1. Table S2. [file NPR2-45-e12512-s001.docx]

**Supplementary Table1**. Distribution of number of tobacco sticks smoked per day

|  | Participants who used cigarettes  No. of participants | Participants who used two products  No. of participants | Participants who used HTPs  No. of participants |
| --- | --- | --- | --- |
| Total | 5208 | 2422 | 3862 |
| 1–10/day | 2741 | 1125 | 2060 |
| 10–20/day | 2005 | 796 | 1279 |
| 20–30/day | 330 | 347 | 331 |
| 30–40/day | 95 | 90 | 94 |
| 40–50/day | 22 | 33 | 32 |
| 50–60/day | 5 | 5 | 22 |
| 60</day | 10 | 26 | 44 |
|  | average, 12  median, 10 | average, 14  median, 11 | average, 16  median, 10 |

**Supplementary Table2**. Multivariable-adjusted odds ratios (95% CIs) for HTPs use in relation to participants’ characteristics: The JASTIS study

|  | No. of HTPs users (%) / No. of all smokers | Odds ratio | 95% confidence interval | P-values |
| --- | --- | --- | --- | --- |
| Gender |  |  |  |  |
| Men | 3350 (58.3) / 5747 | Ref. | Ref. |  |
| Women | 1172 (52.7) / 2222 | 0.80 | 0.72-0.90 | <0.001 |
| Age range (years) |  |  |  |  |
| 20–29 | 1197 (78.1) / 1532 | Ref. | Ref. |  |
| 30–39 | 963 (68.0) / 1416 | 0.49 | 0.41-0.58 | <0.001 |
| 40–49 | 1014 (58.1) / 1746 | 0.33 | 0.28-0.38 | <0.001 |
| 50–59 | 716 (47.3) / 1514 | 0.21 | 0.17-0.24 | <0.001 |
| 60–69 | 496 (42.7) / 1161 | 0.16 | 0.13-0.19 | <0.001 |
| 70–82 | 136 (22.7) / 600 | 0.06 | 0.05-0.08 | <0.001 |
| Household income (yen) |  |  |  |  |
| Less than 2 million | 412 (55.4) / 744 | Ref. | Ref. |  |
| 2–6 million | 1674 (54.3) / 3081 | 0.80 | 0.67-0.96 | 0.014 |
| More than 6 million | 1758 (62.9) / 2794 | 1.00 | 0.83-1.21 | 0.984 |
| Don't know | 377 (49.4) / 763 | 0.89 | 0.71-1.10 | 0.275 |
| Don't want to say | 301 (51.3) / 587 | 0.72 | 0.57-0.92 | 0.007 |
| Alcohol consumption (/day) |  |  |  |  |
| Less than 2 drinks | 2843 (53.7) / 5293 | Ref. | Ref. |  |
| 3–6 drinks | 1357 (61.5) / 2206 | 1.18 | 1.06-1.31 | 0.003 |
| More than 7 drinks | 322 (68.5) / 470 | 1.42 | 1.15-1.76 | 0.001 |
| Education attainment |  |  |  |  |
| Less than high school graduate | 1328 (51.9) / 2560 | Ref. | Ref. |  |
| Vocational school graduate | 839 (54.6) / 1538 | 1.04 | 0.91-1.19 | 0.541 |
| College graduate or higher | 2355 (60.8) / 3871 | 1.20 | 1.07-1.34 | 0.001 |
| Marital status |  |  |  |  |
| Married | 2591 (56.9) / 4556 | Ref. | Ref. |  |
| Never | 1465 (59.0) / 2482 | 0.62 | 0.55-0.70 | <0.001 |
| Divorced/Dead | 466 (50.1) / 931 | 0.92 | 0.79-1.08 | 0.306 |

Odds ratios (95% confidence intervals) were calculated using the logistic regression adjusted for all variables listed.
